# Supplementary material for: Nanoscale 3D DNA tracing in non-denatured cells resolves the Cohesin-dependent loop architecture of the genome in situ
Source: Nat Commun. 2025 Jul 19;16:6673. doi: 10.1038/s41467-025-61689-y (PMC12276220; doi:10.1038/s41467-025-61689-y)
Supplement: Supplementary file 1 — Supplementary Information [file 41467_2025_61689_MOESM1_ESM.pdf]

Supplementary Materials for

Nanoscale 3D DNA tracing in non-denatured cells resolves the Cohesin-dependent loop architecture of the genome in situ

K.S. Beckwith, Ø. Ødegård-Fougner, N.R. Morero, C. Barton, F. Schueder, W. Tang, S. Alexander, J-M. Peters, R. Jungmann, E. Birney, J. Ellenberg

Correspondence to: jan.ellenberg@embl.de

This PDF file includes:

Supplementary Note 1
Supplementary Table S1
Supplementary Figures S1 to S8

Supplementary Note 1: Loop extrusion and Rouse polymer model formulation

Chromatin was modelled with a Rouse polymer using Brownian dynamics similar to previous approaches [1, 2]. The model was initially derived by Rouse from the Langevin motion equation in the over-damped regime:

$$\frac{d}{dt}\boldsymbol{r}_i = \frac{\kappa}{\gamma}[(r_{i-1} - r_i) + (r_{i+1} - r_i)] + \sqrt{6D}\frac{d\xi}{dt}$$

Where $\mathbf{r}_\mathrm{i}$ is the position of the ith bead, κ is the spring constant of the Hookean spring coupling the beads, γ is the friction coefficient, D is the diffusion coefficient of each bead, while ξ is a Gaussian noise with zero mean and unit variance. The rouse polymer was simulated in an Euler scheme:

$$r_i(t + \Delta t) = r_i(t) + \frac{\kappa}{\gamma}[(r_{i-1} - r_i) + (r_{i+1} - r_i)] + \sqrt{6D\Delta t}\xi$$

with $\Delta t = 0.01s$, and each bead a monomer of 1 kb. There are numerous estimates of the diffusion coefficient of chromatin depending on experimental approaches. We chose an intermediate value [3] for our simulation and swept $\frac{\kappa}{\gamma}$ values until reaching a polymer simulation showing physical distance scaling similar to high resolution tracing of 300 kb regions in cohesin-depleted cells (r^2= 0.996 for MEOX1 region). The values for all simulation parameters are listed in Supplementary Table 1. In addition, a hard-shell potential set at 20 nm was used between interacting beads to avoid spatial overlap. Loop extrusion was simulated by adding an extra spring between non-adjacent monomers corresponding to the left and right sides of a single stepping Cohesin, with the same spring constant as between adjacent monomers. Cohesin was modeled as two populations (Cohesin-STAG1 and Cohesin-STAG2), with their experimentally measured cellular abundances [4] and adjusted to per-Mb values, distributed proportionally onto the simulated polymer, and off-rates corresponding to residence times matching kinetic FRAP data of STAG1 and STAG2 in CTCF-depleted cells [5]. On-rates were set so that the average bound fraction matched experimental data [5]. Upon binding, each Cohesin was placed at a random position on the polymer and while bound each side stepped left and right, respectively, along the polymer with a given rate (extrusion rate). If one side stepped outside the polymer, the Cohesin was unbound.

The boundary factor CTCF was modelled with an off-rate from the residence time measured from FRAP and single particle tracking experiments [6]. Experimentally measured cellular abundances of CTCF [4] were distributed among all CTCF Chip-seq sites with annotated motif orientations [7]. Upon binding, each CTCF would target one of the peak sites in the simulated region with a probability matching the relative signal of the Chip-seq peak in the simulated region. We note that this simplified approach of distributing CTCFs may lead to occasional discrepancy between the model and experimental data, as CTCF boundary strength and Chip-seq signal magnitude are not always directly correlated [8].

At each timestep, binding or unbinding of Cohesin and CTCF occurred with a probability of the inverse of the respective residence time, giving an exponentially distributed lifetime corresponding to the FRAP or single molecules measurements [5, 6].

We made the following assumptions of the interactions between CTCF and Cohesin based on recent studies [4, 5, 9, 10]: If a Cohesin encountered a CTCF in a convergent orientation, the respective side was stalled as long as the CTCF remained bound, and a new, extended lifetime in the CTCF-bound state was used [5]. CTCF residence time remained unchanged [6]. Each CTCF could bind one Cohesin, while

each Cohesin could bind two CTCFs, one with each side, and remained stalled on the respective side until the corresponding CTCF unbound. No interactions between Cohesins were included in the model.

Loop extrusion simulations were performed by adding Cohesins and CTCFs in an unbound state and equilibrated with 2000 initiation loop-extrusion timesteps (1 second steps), before stochastically halting before 4000 simulation steps were reached. To reduce boundary effects, loop extrusion simulations were performed on a genomic region padded by 2 Mb on each side of the targeted genomic region. The resulting Cohesin loops were used to simulate a Rouse polymer.

Each Rouse polymer simulation was initialized from a random walk with a step size standard deviation of $\sqrt{6D}$ of a region padded with 100 kb on each side of the genomic target, and pre-equilibrated with 10 000 timesteps (0.01 s) before adding the Cohesin loops and running for another 10 000 steps. Each loop extrusion and corresponding polymer simulation was repeated to generate 3000 simulated traces. Gaussian noise giving rise to a median 50 nm 3D error was added to simulate conservative experimental conditions.
After calibration against tracing data from Cohesin and CTCF-depleted HeLa cells (see main text), a reduced abundance of Cohesins with a 1kb/s extrusion rate were used for the remainder of the simulations, which were further used to constrain effective CTCF abundances.

ΔWAPL simulations were performed by setting the Cohesin-STAG1 residence time to the same value as the CTCF-bound lifetime. 5000 initiation steps and up to 5000 running steps (average 2500) were used in the loop extrusion simulation to approximate a 2h WAPL depletion. All simulation code is available as part of the LoopTrace package (see Code availability).

Supplementary Table S1: Simulation parameters used for loop extrusion simulations

Parameter	Value	Reference
D	3.4e-3 μm^2/s	[3]
$\frac{\kappa}{\gamma}$	0.13	Estimated from HeLa ΔRAD21 measurements (this work).
CTCFs per Mb	Variable, see text.	Initial estimate from [4], adjusted to achieve Cohesin-STAG1 bound fraction of ~0.8.
Total Cohesin per Mb	Variable, see text.	Initial estimate from [4], adjusted to match HeLa ΔCTCF data.
Cohesin-STAG1/STAG2 ratio	0.2	[4]
Cohesin-STAG1 CTCF-free bound fraction	0.5	[5]
Cohesin-STAG1 bound fraction with CTCF	0.8	[5]
Cohesin-STAG1 chromatin bound lifetime	900 s	[5]
Cohesin-STAG1 CTCF bound lifetime	18 000 s	[5]
Cohesin-STAG1 unbound lifetime	900 s	Estimated from bound fraction
Cohesin-STAG2 CTCF-free bound fraction	0.45	[5]
Cohesin-STAG2 bound fraction with CTCF	0.5	[5]
Cohesin-STAG2 chromatin bound lifetime	480 s	[5]
Cohesin-STAG2 CTCF bound lifetime	900 s	[5]
Cohesin-STAG2 unbound lifetime	620 s	Estimated from bound fraction
Cohesin extrusion rate	1 kb/s (0.5 kb/s per side)	Initial estimates from [9], empirically adjusted in this work.
CTCF bound fraction	0.5	[6]
CTCF chromatin bound lifetime	120 s	[6]
CTCF unbound lifetime	120 s	[6]

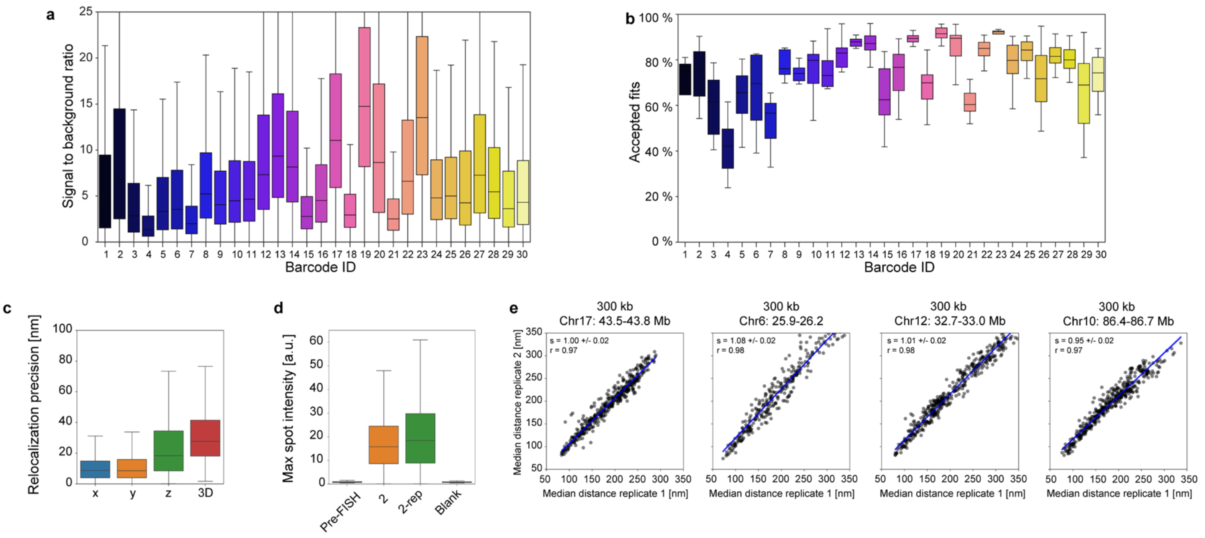

Supplementary Figure S1

a, Signal to background ratio of individual 12-mer barcodes targeting groups of 50-100 oligopaint probes spanning 10 kb. Data pooled from four genomic regions. **b,** Percentage of fits for each barcode passing quality control thresholds from four 300 kb genomic regions. Average barcode fitting efficiency was 79%. **c**, Tracing precision in x, y, z and 3D measured by difference in centroid position of 10 kb spots re-labelled after 30 exchange cycles. **d**, Maximum signal intensity in tracing ROIs in the indicated imaging cycle. **e,** Correlation between median pairwise distances in replicate experiments of four 300 kb regions using the non-denaturing FISH approach determined by linear regression. Scaling coefficient (s) and Pearson's correlation (r) are indicated in each plot. Pearson's correlation coefficient from the four genomic regions was r=0.975±0.006 (mean±std). Boxplots in **a-d** show median values (horizontal line), lower and upper quartiles (box) and values within 1.5 times interquartile range (IQR, whiskers). **a**, **d** and **e**, data from two independent experiments with n=1845 traces in RPE-1 cells. **b** and **c**, data from n=663 traces in one representative experiment and genomic region in RPE-1 cells. Source data are provided as a Source Data file.

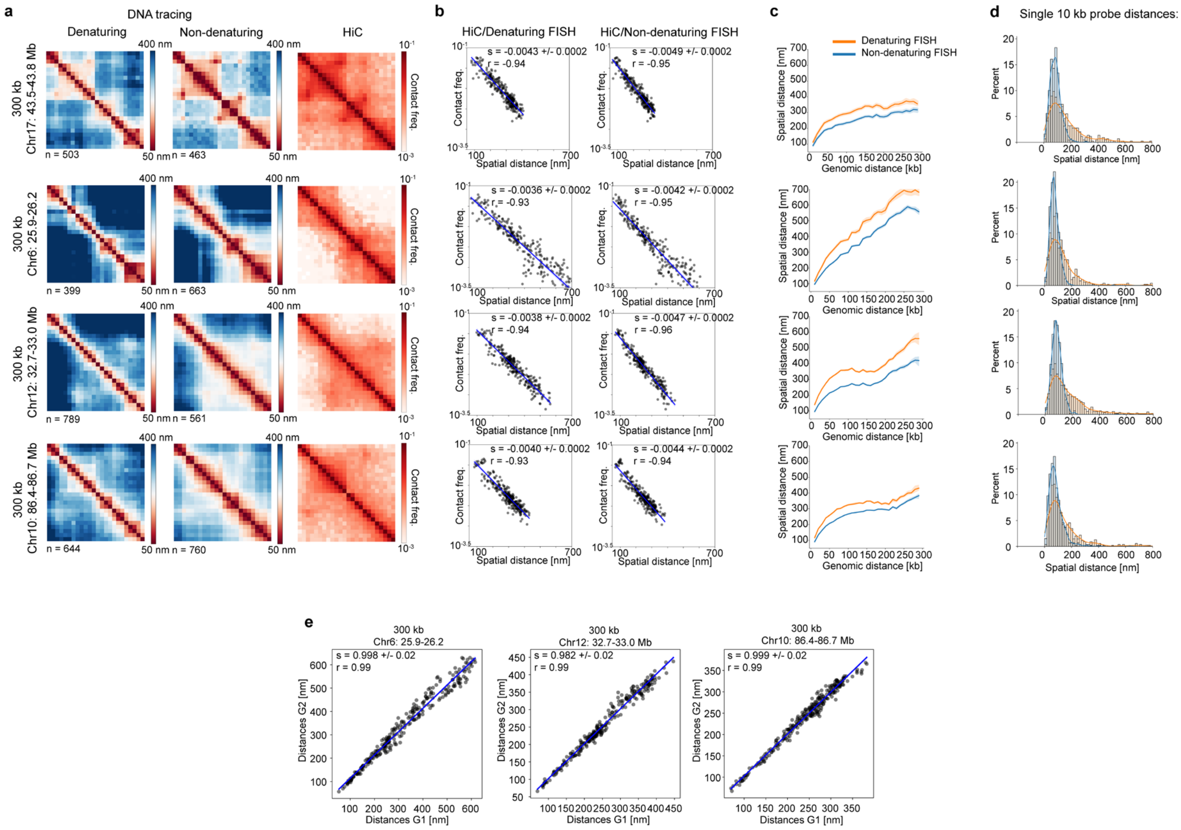

Supplementary Figure S2

a, Median pairwise distance maps of four 300 kb regions traced at 10 kb resolution in RPE-1 cells with denaturing (left column) and non-denaturing (middle column) FISH approaches and HiC contact frequencies (right columns) from publicly available RPE-1 HiC data (GSE71831). Only cells assigned to G1 phase by DAPI intensity were used. **b,** Correlation between median pairwise distances measured by each DNA tracing approach and log-transformed contact frequencies by HiC. Slope (s) and Pearson's r (r) from a linear regression (blue line) are indicated. **c,** Physical distance scaling of the four 300 kb genomic regions with the two FISH approaches. Shaded areas indicate 95% confidence interval of the median line. **d,** Representative pairwise distance distributions between two consecutive 10 kb positions for the two FISH approaches, displayed with a kernel density estimate overlaying the histogram. **e,** Correlation between median pairwise distances from the additional 300 kb regions of cells separated into G1 and G2 populations with the non-denaturing FISH approach. Slope (s) and Pearson's r (r) from a linear regression (blue line) are indicated. Number of traces analysed from each region indicated in **a,** and the data is representative of two independent experiments per condition. Source data are provided as a Source Data file.

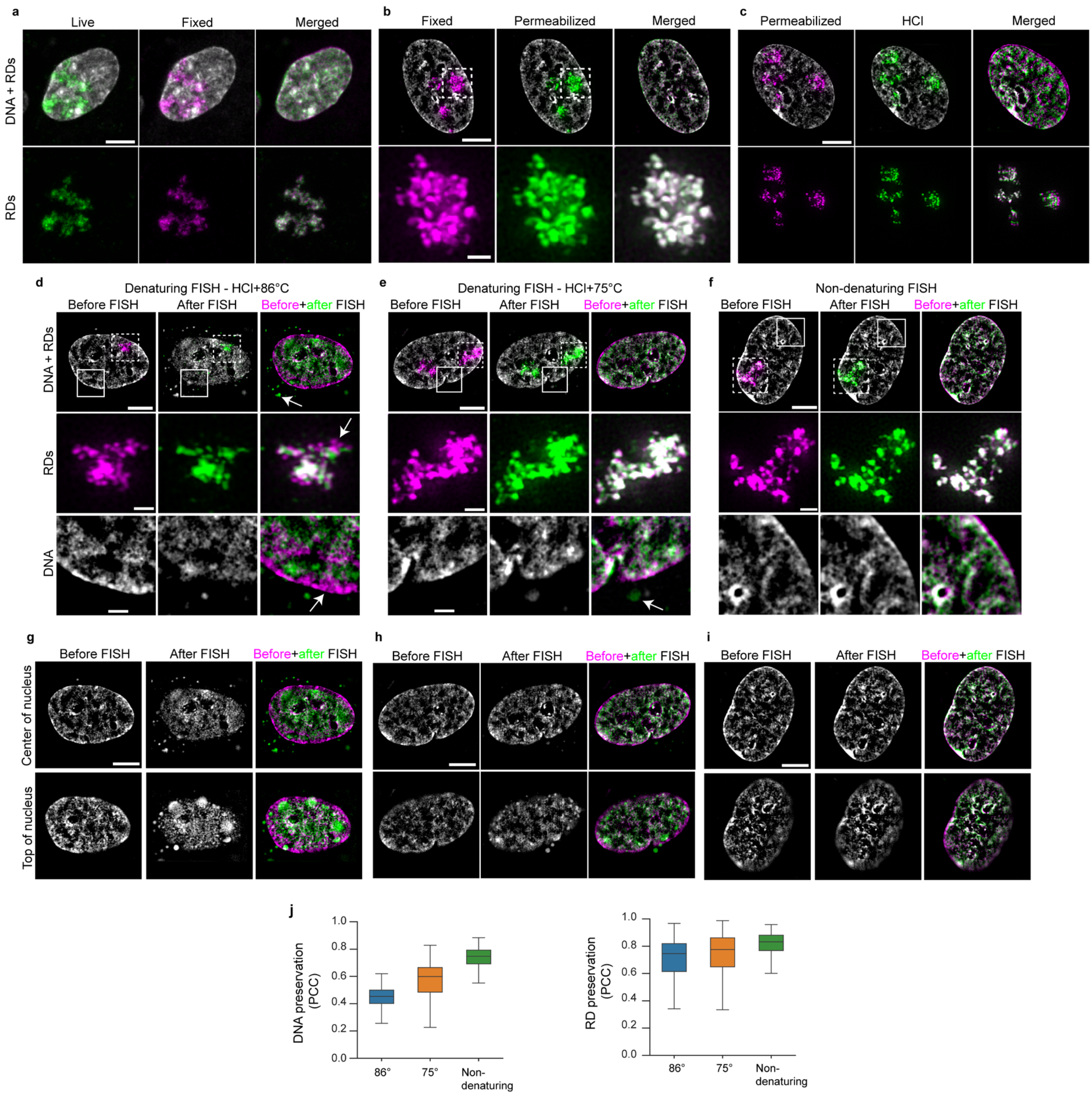

Supplementary Figure S3

Comparison of preservation of chromatin structure during individual steps of the FISH protocol assessed by confocal imaging (**a**) or structured illumination microscopy (SIM, **b-i**) of RPE-1 cells labelled with Hoechst (DNA, grey) and Atto647-dUTP (replication domains, RDs, magenta/green).: **a,** from live to fixed cells; **b,** from fixed to permeabilized cells; and **c,** from permeabilization to HCl treatment. Images representative of >10 (**a**) or >50 cells (**b, c**) in two independent experiments. **d-f,** Structural preservation of nuclear architecture after alternative FISH protocols. Extra-nuclear DNA material and shifts in RD position indicated by arrows. Single z-planes are shown. **h-i,** Comparison of central and apical planes of cell nuclei before and after FISH treatment for the conditions described in **d-f**. **j,** Pearson's correlation of signal intensity of DAPI (left panel) and RDs (right panel) in 3D drift-corrected SIM images before and after FISH. Boxplots show median values (horizontal line), lower and upper quartiles (box) and values within 1.5 times IQR (whiskers). **d-j,** Data from n > 350 cells per condition from two independent experiments. Scale bars, 5 µm; RDs in **b; d-f**, 1 µm. Source data are provided as a Source Data file.

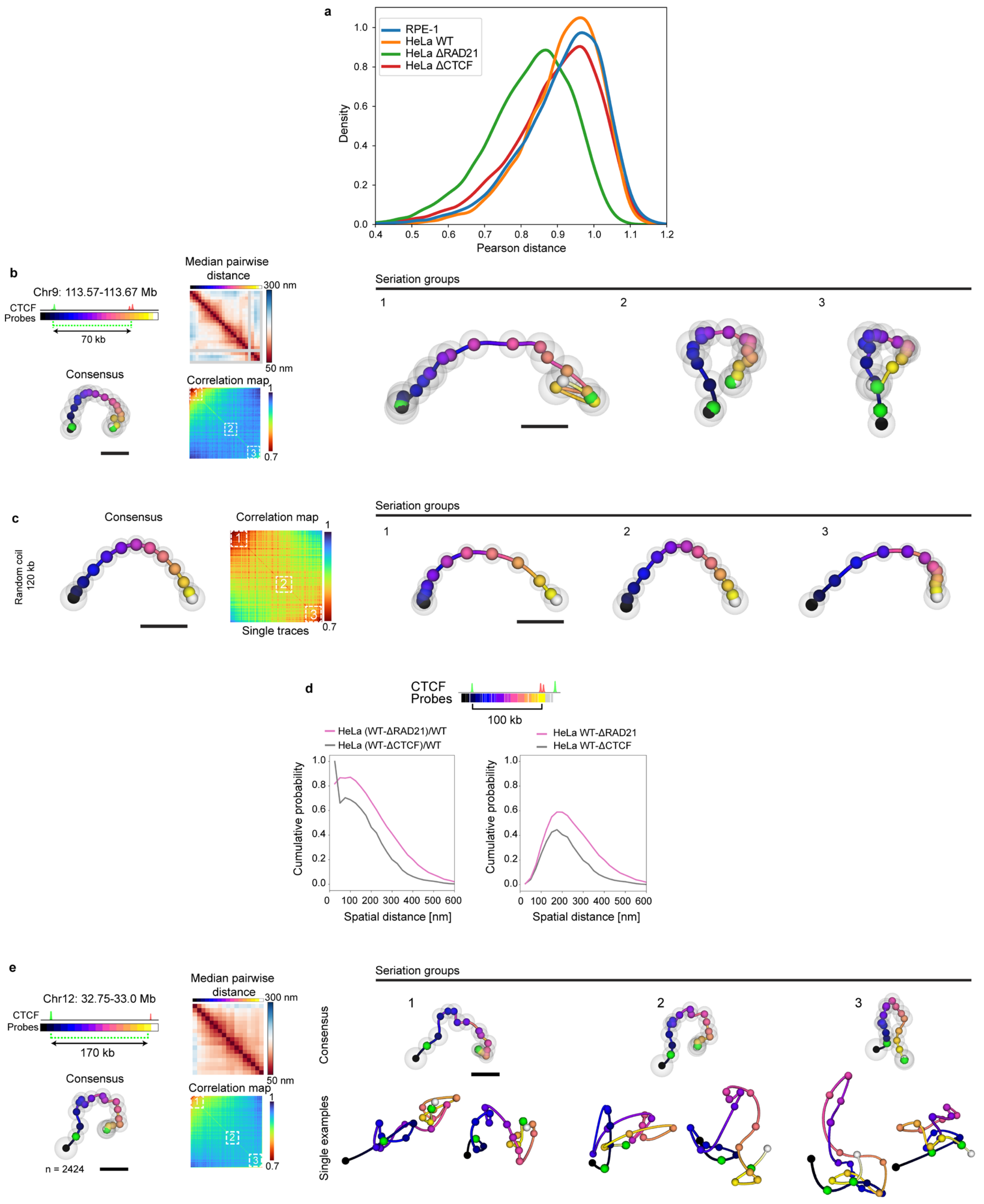

Supplementary Figure S4

a, Pairwise Pearson's correlations for individual traces from the loop domain near *MEOX1* at Chr17:43.6-43.7 Mb from data described in Main Fig. 3. **b,** Overview of the genomic region at Chr9:113.57-113.67, median pairwise distance map, consensus trace, pairwise Pearson correlation map and consensus traces from seriation groups for a 100 kb region traced at 4 kb resolution in RPE-1 cells. n=1641 traces from two independent experiments. Positions of CTCF sites from Chip-seq labelled with green spheres, grey spheres indicate standard deviations of all traces compared to the consensus. **c,** Consensus traces, correlation map and consensus traces of seriation groups of simulated random coils. **d,** Relative (left) and absolute (right) difference in probability of an interaction at a

given distance between the convergent CTCF sites near *MEOX1* on Chr17 in WT compared to Cohesin or CTCF-depleted cells, suggesting that 100 nm is the most selective cut-off for a Cohesin and CTCF-specific interaction at this genomic distance. n=2349 traces from two independent experiments. **e,** Overview of the 190 kb genomic region at Chr12:32.8-33.0, median pairwise distance map, consensus trace, pairwise Pearson correlation map and consensus traces and individual trace examples from the indicated seriation groups. Grey spheres indicate the standard deviation of each position compared to the consensus representation. n=1753 traces from two independent experiments. Source data are provided as a Source Data file.

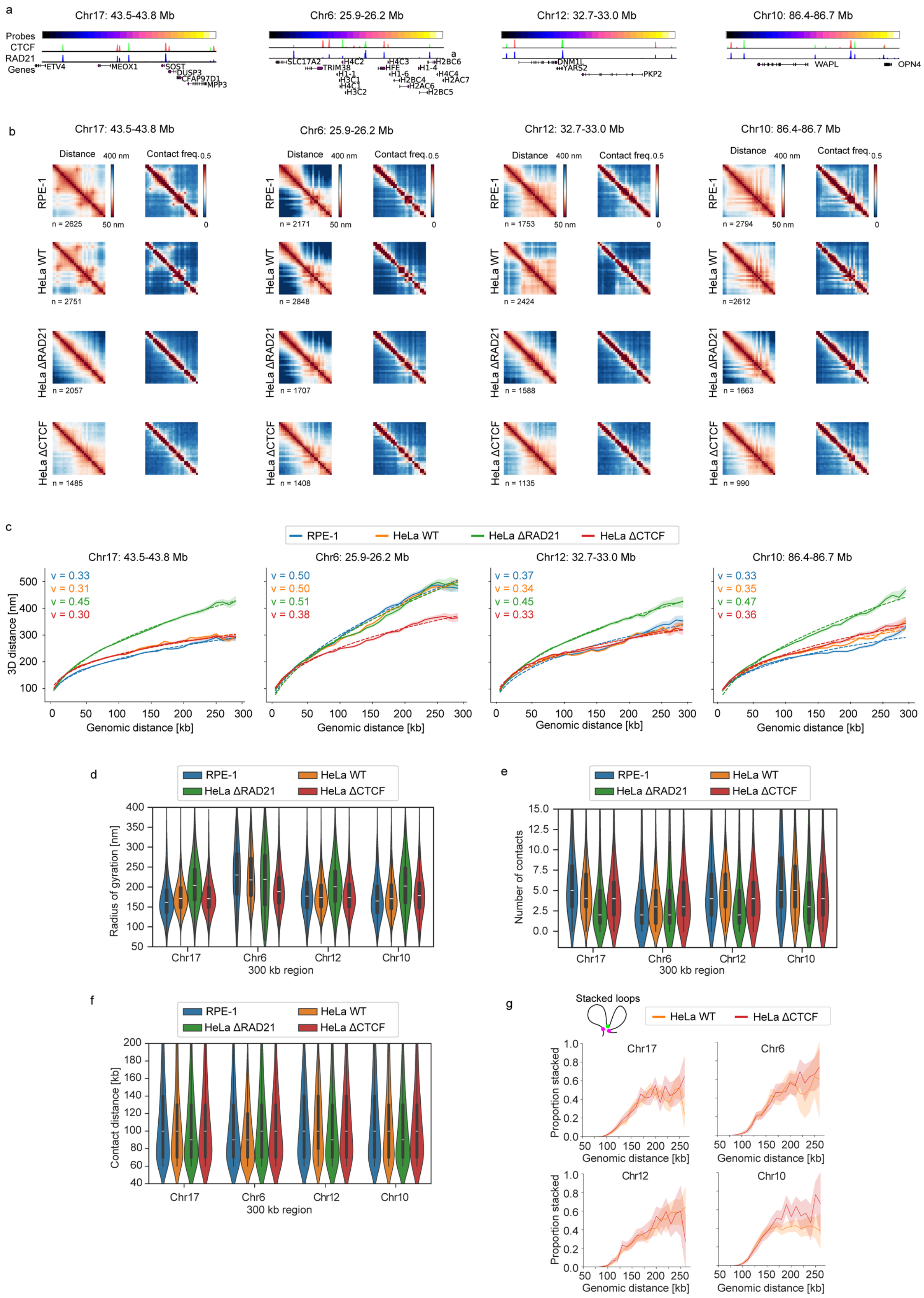

Supplementary Figure S5

a, Genomic overview of four 300 kb regions traced at 10 kb resolution, indicating probe positions, CTCF chip-seq peaks with color-coded orientation, Rad21 chip-seq peaks and gene annotations. **b,**

Median 3D distance scaling, from four 300 kb genomic regions traced at 10 kb resolution in the indicated cells lines. Number of traces per condition indicated in figure. Data from two independent experiments per condition. **c,** Median 3D distance scaling from the indicated regions and cell lines corresponding to the data in **b**. Scaling coefficients from a power law fit ($d_{spatial} = (d_{genomic})^{v}$) are labelled and the resulting fits shown as dashed lines. Shaded areas indicate 95% confidence interval of the median line. **d-g,** Radius of gyration, number of contacts, genomic distance between contacts and proportion of stacked contacts (contacts spanning a longer genomic distance that can be subdivided into two or more sets of coinciding contacts) from the four regions and cell lines shown in **b.** Violin plots in d-f show median values (white line), lower and upper quantiles (boxes), values with 1.5 times IQR (whiskers) and the data range (violins). Shaded areas in **g** indicate 95% confidence interval of the median line. Source data are provided as a Source Data file.

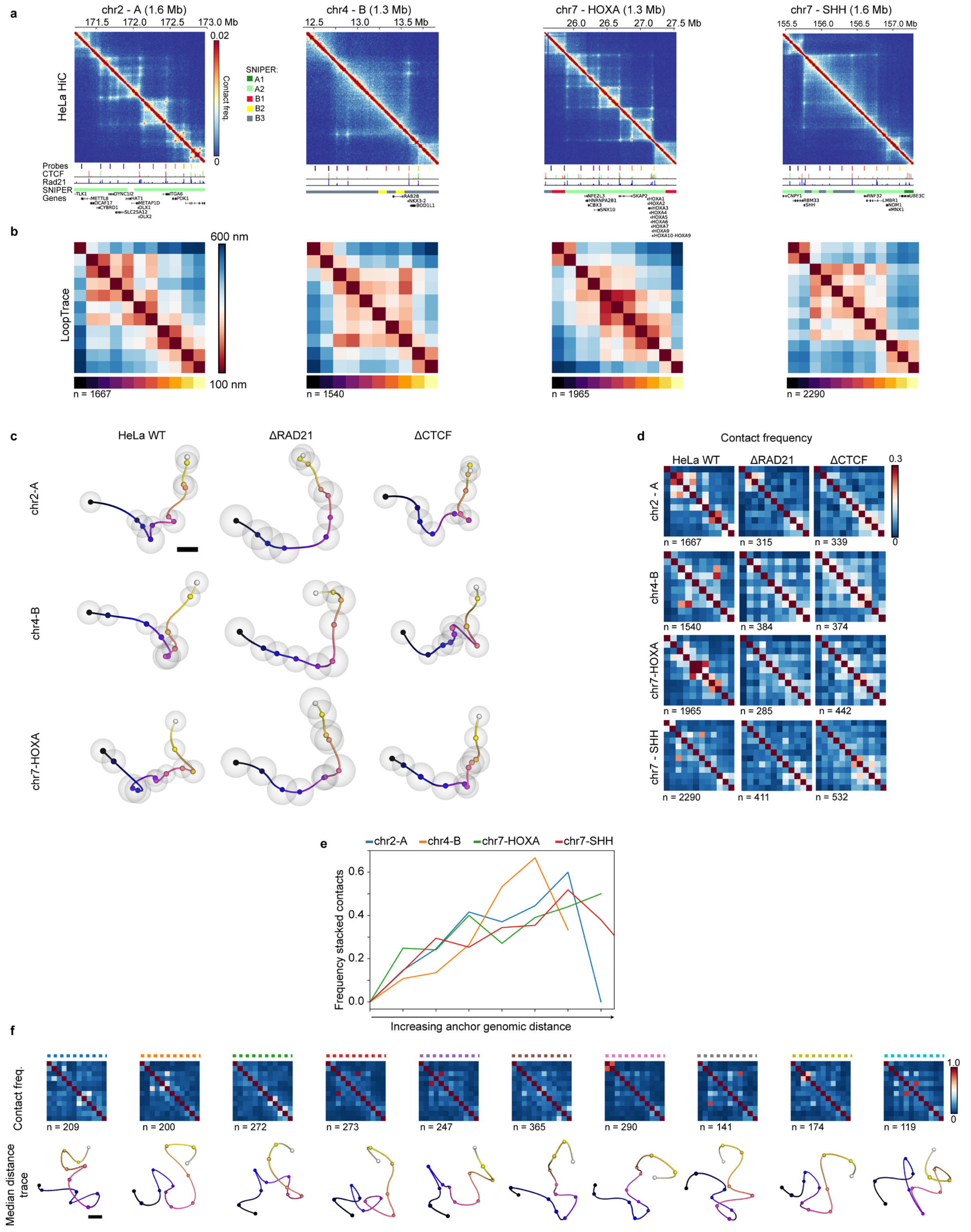

Supplementary Figure S6

a, HeLa HiC maps binned at 10 kb of the four TAD-scale regions selected for tracing. The probes, as well as HeLa CTCF ChIP-seq peaks indicating motif directionality, HeLa Rad21 ChIP-seq peaks and SNIPER compartmental annotations are listed. **b,** Median 3D pairwise distance maps of measured probe positions in the four indicated regions in HeLa cells. Grey spheres indicate the standard deviation of each position compared to the consensus representation. Trace numbers as indicated from three independent experiments. **c,** Consensus traces from HeLa WT cells or HeLa Rad21-mEGFP-AID or

CTCF-mEGFP-AID cells treated with auxin for 2 h. **d**, Contact frequencies of the regions and treatment conditions in (**c**). **e,** Frequency of longer-range contacts established by stacking of shorter contacts, sorted according to distance spanned by the largest contact. **f**, Contact frequencies and median distance reconstructions of the clusters in Main Fig. 5d indicated by coloured dashed lines**.** Data from three (wild type) or two (AID lines) experiments, number of traces per group as indicated. Scale bars 100 nm. Source data are provided as a Source Data file.

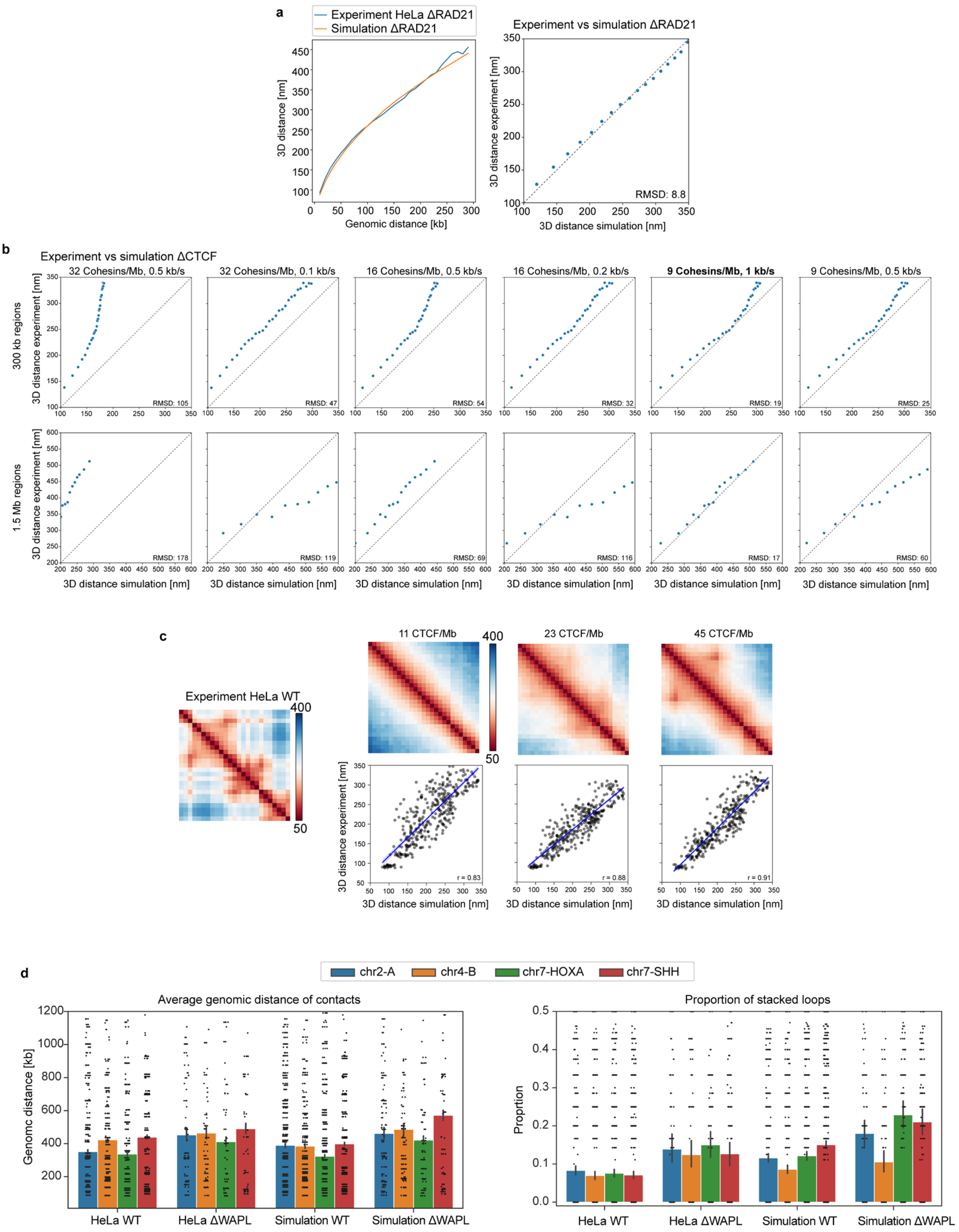

Supplementary Figure S7

a, Median 3D distance scaling (left) and scatter plot of experimental and simulated median distances (perfect correlation is indicated by dashed line, right). Experimental data pooled from four 300 kb genomic regions in HeLa cells depleted of Cohesin. The best fit (lowest RMSD) after sweeping spring constants is shown. **b**, Scatter plots of simulated 3D distances and median experimental 3D distances from HeLa cells depleted of CTCF from four pooled 300 kb (top) or ~1.5 Mb (bottom) regions. The number of Cohesins per Mb were swept until the lowest RMSD for both regions were reached (indicated

in bold). Perfect correlation is indicated by dashed line **c**, Median pairwise distance map for experimental HeLa WT data for Chr17:43.5-43.8 (right) and the corresponding region from the simulation (right). Increasing number of CTCF molecules were added to the simulations until the best fit (r=0.89) with the data was obtained. **d,** Quantification of genomic length of non-stacked contacts and proportion of emergent loops formed by stacking two or more shorter loops. Experimental data from HeLa WT cells and HeLa WAPL-Halo-AID treated for 2h with auxin and corresponding polymer simulations from four 1.4-1.8 Mb genomic regions traced with 10-12 probes targeting CTCF sites and intermediate regions. Mean and 95% confidence interval of mean shown with single data points shown as black markers. n=3887 traces from three independent experiments in WT cells and n=555 traces from two independent experiments in WAPL-depleted cells. Source data are provided as a Source Data file.

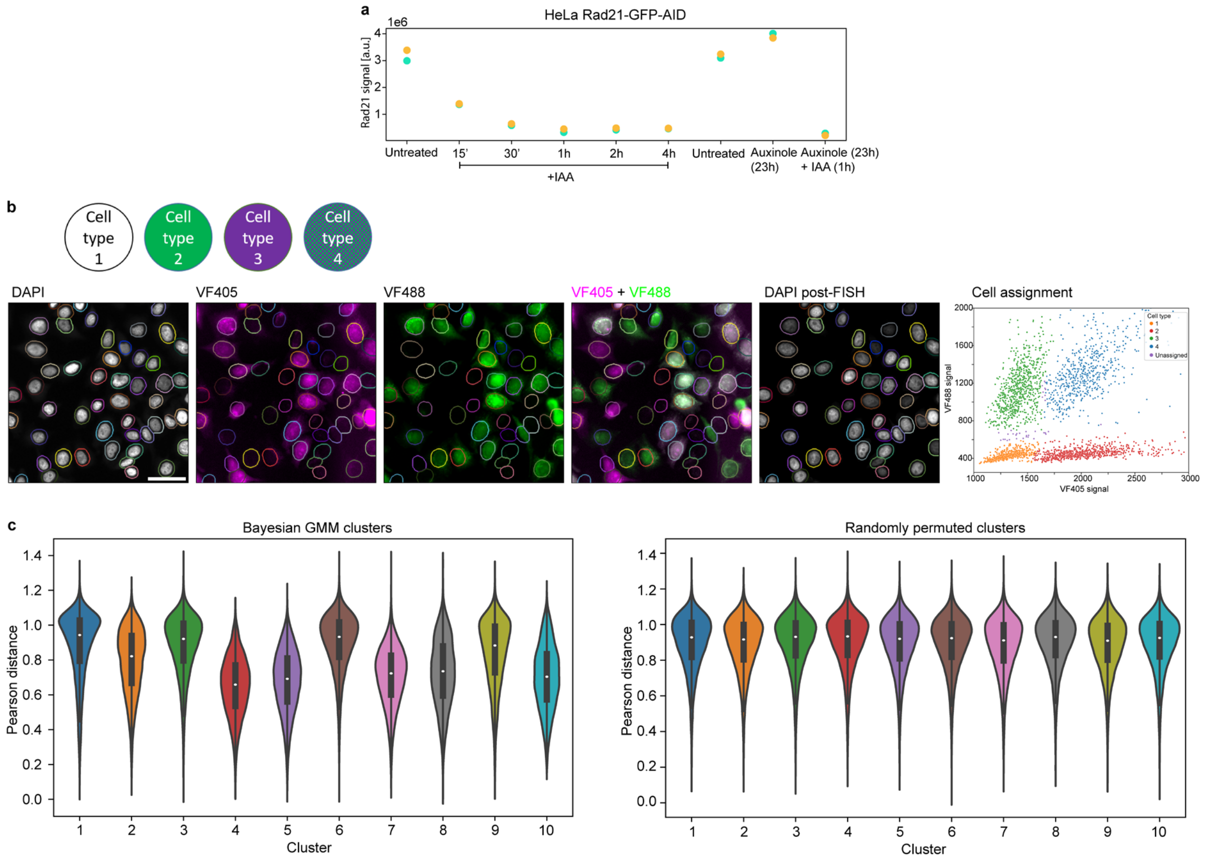

Supplementary Figure S8

a, Chemiluminescence signal from simple Western assay using anti-Rad21 in untreated HeLa Rad21-mEGFP-AID cells or treated with auxin (IAA), auxinole or both for the indicated durations. Yellow and cyan markers indicate technical duplicates from one experiment. b, Overview of barcoding strategy to simultaneously acquire data from multiple cell lines. Each of up to four cell lines is either left unlabelled, or pre-labelled with VF405, VF488 or both before mixing and seeding the cells in the sample chamber. After fixation, VF405 and VF488 signals in cells in the entire sample chamber are imaged at lower magnification, then labelled with DAPI and reimaged before being processed for FISH. The same cells are relocated by registering the DAPI channel acquired during sequential FISH imaging with the pre-FISH images, and detecting the VF405 and VF488 intensities in a dilated nuclear mask (pseudo-coloured ellipses). The original cell types are then recovered by a manual gating strategy based on the VF405+VF488 intensity. Images representative of 100 fields of view in two independent experiments. c, All pairwise intra-cluster differences (using $\sqrt{(1\text{-PCC})}$ as distance metric) of traces from the Chr7-SHH region assigned to clusters shown in Main Fig. 5d and Supplementary Fig. S6f (left), compared with randomly permuted cluster assignments (right). Overall differences detected by comparing the means of the real and permuted clusters ($p=0.001$, two-tailed t-test, total of $n=2290$ traces in 10 clusters). Results were identical for repeated permutations. Source data are provided as a Source Data file.

Supplementary References

1. Fudenberg, G., et al. *Formation of Chromosomal Domains by Loop Extrusion*. 2016.
2. Sanborn, A.L., et al., *Chromatin extrusion explains key features of loop and domain formation in wild-type and engineered genomes.* Proceedings of the National Academy of Sciences, 2015. **112**: p. E6456 - E6465.
3. Barth, R., K. Bystricky, and H.A. Shaban, *Coupling chromatin structure and dynamics by live super-resolution imaging.* Science Advances, 2020. **6**(27): p. eaaz2196.
4. Holzmann, J., et al., *Absolute quantification of cohesin, CTCF and their regulators in human cells.* eLife, 2019. **8**.
5. Wutz, G., et al., *ESCO1 and CTCF enable formation of long chromatin loops by protecting cohesinSTAG1 from WAPL.* eLife, 2019. **9**.
6. Hansen, A.S., et al., *CTCF and cohesin regulate chromatin loop stability with distinct dynamics.* eLife, 2016. **6**.
7. Rao, S.S.P., et al., *A 3D Map of the Human Genome at Kilobase Resolution Reveals Principles of Chromatin Looping.* Cell, 2014. **159**: p. 1665-1680.
8. Nanni, L., S. Ceri, and C. Logie, *Spatial patterns of CTCF sites define the anatomy of TADs and their boundaries.* Genome Biology, 2020. **21**(1): p. 197.
9. Rao, S.S.P., et al., *Cohesin Loss Eliminates All Loop Domains.* Cell, 2017. **171**: p. 305-320.e24.
10. Wutz, G., et al., *Topologically associating domains and chromatin loops depend on cohesin and are regulated by CTCF, WAPL, and PDS5 proteins.* The EMBO Journal, 2017. **36**: p. 3573 - 3599.
