# Supplementary material for: Nanoscale 3D DNA tracing in non-denatured cells resolves the Cohesin-dependent loop architecture of the genome in situ
Source: Nat Commun. 2025 Jul 19;16:6673. doi: 10.1038/s41467-025-61689-y (PMC12276220; doi:10.1038/s41467-025-61689-y)
Supplement: Supplementary file 3 — Description of Additional Supplementary Files [file 41467_2025_61689_MOESM3_ESM.pdf]

Description of Additional Supplementary Files

File Name: Supplementary Data 1
Description: Oligonucleotide sequences.
